# Evaluating Convolutional and Transformer Architectures for Photovoltaic Defect Classification via Electroluminescence Imagery

**DOI:** 10.3390/s26123775

**Published:** 2026-06-13

**Authors:** Seda Bayat Toksöz, Gültekin Işık, Gökhan Şahin, Erdal Akin

**Affiliations:** 1 Department of Computer Engineering, Iğdır University, Iğdır 76000, Türkiye; 2 Municipality of Dronten, De Rede, 1, 8251 ER Dronten, The Netherlands; 3 Copernicus Institute of Sustainable Development, Utrecht University, Princetonlaan 8A, 3584 CB Utrecht, The Netherlands; 4 Department of Computer Science and Media Technology, Malmö University, 205 06 Malmö, Sweden; 5 Sustainable Digitalisation Research Centre, Malmö University, 205 06 Malmö, Sweden; 6 Biofilms Research Center for Biointerfaces (BRCB), Malmö University, 205 06 Malmö, Sweden; 7 Department of Computer Engineering, Bitlis Eren University, Bitlis 13100, Türkiye

**Keywords:** photovoltaic defect classification, electroluminescence imaging, convolutional neural network, vision transformer, ConvNeXt, stratified cross validation, parameter efficiency

## Abstract

Electroluminescence (EL) imaging is widely used for photovoltaic (PV) defect inspection, yet fair comparison of deep learning backbones remains difficult because datasets, labels, and protocols vary across studies. This work presents a controlled image-level benchmark of six architectures (ConvNeXt-T, ViT-B/16, DeiT-B/16, Swin-T, DenseNet121, and MobileNetV3-Large) across five hierarchical tasks for monocrystalline and polycrystalline cells with binary and multi-class labels. A balanced proprietary dataset of 20,000 single-cell EL images was evaluated with identical preprocessing, augmentation, training, and stratified five-fold cross-validation, yielding 150 runs. ConvNeXt-T achieved the highest mean macro-F1 (93.12%) while using about one-third of the parameters of base ViT/DeiT models. On the four-class polycrystalline task, it reached 84.94 ± 0.45% macro-F1, compared with 70.08 ± 1.19% for DenseNet121 and 59.43 ± 1.71% for MobileNetV3-Large. Error analysis revealed conservative missed-defect behavior in lightweight CNNs, especially for surface-level degradation and crack categories. The results provide image-level cross-validation evidence for controlled benchmarking and motivate future module-level grouped validation.

## 1. Introduction

The growing global demand for sustainable and low-carbon energy has established photovoltaic (PV) systems as a central component of modern energy infrastructure [1,2]. The performance, reliability, and service life of PV modules are strongly affected by the structural integrity of individual solar cells [3]. Defects such as microcracks, broken fingers, inactive regions, surface degradation, and material irregularities can occur during manufacturing, handling, transportation, and field operation, leading to power loss and reduced module lifetime [4,5]. Early and accurate defect identification is therefore essential for quality assurance, reliability monitoring, and cost-effective maintenance [6].

Electroluminescence (EL) imaging is particularly suitable for this purpose because it reveals subsurface and electrically inactive regions that are difficult to detect using conventional visual inspection [7,8,9].

Automated EL inspection has increasingly moved from handcrafted feature extraction toward deep learning. CNN-based models provide strong local inductive biases and have achieved high accuracy in PV defect classification and segmentation [9,10,11,12]. Transformer-based models can capture long-range contextual relationships, but their larger computational and data requirements may limit their advantage in industrial EL settings [13,14,15,16,17]. More recent hybrid CNN–transformer approaches attempt to combine locality and global context; however, many PV studies still differ in dataset composition, label granularity, splitting strategy, and metric reporting, which makes direct architectural comparison difficult. The novelty of this study is therefore deliberately positioned as a benchmarking and evaluation contribution rather than as a new classifier architecture. We ask whether modern convolutional, transformer, hierarchical transformer, and lightweight CNN backbones remain comparable when they are trained under the same preprocessing, augmentation, optimization, and cross-validation protocol. This framing is important because a model that performs well on a single train–test split or on a simplified binary task may not remain stable under finer defect taxonomies, repeated folds, or missed-defect evaluation.

This study makes four main contributions. First, it provides a unified five-task benchmark over 20,000 single-cell EL images, covering material type recognition, binary defect detection, and fine-grained mono- and polycrystalline defect classification. Second, it compares six representative architectures under identical training and evaluation conditions, producing 150 independently trained models. Third, it reports not only aggregate macro-F1 but also fold stability, precision–recall asymmetry, and per-class recall to expose missed-defect behavior. Fourth, it explicitly discusses reproducibility and validity constraints, including proprietary data access and the distinction between image-level stratified cross-validation and stricter module-level grouped evaluation. The remainder of this manuscript is organized as follows. Section 2 reviews previous studies on EL-based defect detection and summarizes the selected deep learning architectures. Section 3 explains the dataset, experimental setup, and evaluation methodology. Section 4 presents the experimental results together with statistical analyses. Section 5 discusses the implications of the findings, common error patterns, and study limitations. Finally, Section 6 concludes the paper.

## 2. Related Work

### 2.1. Deep Learning for EL-Based PV Defect Detection

Reference [8] introduced one of the earliest and most widely used electroluminescence imaging datasets, namely ELPV, together with a CNN-based baseline for defect classification. Subsequent studies extended this direction using lightweight CNNs, deeper residual networks, attention modules, GAN-based augmentation, and ensemble strategies [18,19,20,21,22,23]. These works demonstrate the practical value of deep learning for PV inspection, but many are evaluated using a single train–test split or simplified binary/coarse-grained labels. More recent work has also explored hybrid designs, including CNN-EML and CNN–transformer approaches for EL-based PV fault diagnosis [24,25]. These developments motivate a controlled comparison between modern convolutional, hierarchical transformer, base transformer, and lightweight architectures under a common protocol. The present study addresses this gap by evaluating six backbones across five hierarchical tasks with identical preprocessing, optimization, and stratified five-fold cross-validation.

### 2.2. Architectures Considered in This Study

DenseNet121 [26] was included as a classical CNN baseline because dense interlayer connectivity encourages feature reuse and parameter efficiency. MobileNetV3-Large [27] represents a mobile-oriented CNN family designed for resource-constrained deployment. ViT-B/16 [13] was selected as a plain transformer baseline that replaces convolutional inductive biases with patch-wise self-attention. DeiT-B/16 [28] provides a data-efficient transformer variant trained with distillation-based strategies, while Swin-T [15] represents a hierarchical transformer with shifted local windows. ConvNeXt-T [29] was included as a modern purely convolutional architecture that incorporates several design principles associated with hierarchical transformer models, including larger receptive fields, inverted bottlenecks, and LayerNorm-based normalization. Together, these models cover the main architectural families relevant to EL-based PV inspection: conventional CNNs, mobile CNNs, modern CNNs, base vision transformers, hierarchical transformers, and distillation-enhanced transformers.

These six architectures were deliberately selected to represent contrasting inductive biases that are particularly relevant to the subtle and localized defect patterns observed in EL imagery. DenseNet121 benefits from dense interlayer connectivity and feature reuse, which is advantageous for capturing fine-grained crack and surface textures. MobileNetV3-Large evaluates the feasibility of lightweight deployment in resource-constrained industrial inspection pipelines. ViT-B/16 and DeiT-B/16 test the effectiveness of global self-attention mechanisms for modeling long-range dependencies such as crack propagation across the cell. Swin-T introduces hierarchical processing with shifted local windows, naturally aligning with multi-scale defect morphologies. Finally, ConvNeXt-T tests whether a modern purely convolutional architecture that adopts several transformer-inspired macro-design principles, including larger receptive fields, inverted bottlenecks, and LayerNorm-based normalization, can deliver a favorable accuracy–efficiency trade-off for EL-based PV defect classification.

## 3. Materials and Methods

This section describes the dataset construction process, defect taxonomy, task hierarchy, preprocessing pipeline, model configurations, training strategy, and evaluation methodology. Each component was chosen to isolate architectural effects as much as possible. The same single-cell EL image repository was used across all tasks, the same preprocessing and augmentation policies were applied to every backbone, and identical cross-validation folds were reused for all models within a given task. Consequently, differences in performance can be interpreted primarily as differences in model behavior rather than artifacts of different data splits, preprocessing choices, or training schedules.

No chemicals, reagents, biological materials, commercial cell lines, or laboratory consumables were used in this study. The study was conducted as a computational benchmark based on pre-acquired electroluminescence images of crystalline silicon photovoltaic cells. The photovoltaic cell/module image samples were provided by an industrial data partner; however, the manufacturer, model, and production location of the original commercial photovoltaic cells/modules were not disclosed under the data-sharing agreement. Similarly, the electroluminescence image acquisition was performed by the industrial data provider using a silicon charge-coupled device camera, a darkroom acquisition environment, and a forward-bias electrical excitation unit; the manufacturer/model information of the original camera, power supply, and darkroom system was not disclosed to the authors. Therefore, these acquisition-device and commercial-sample details are explicitly reported as undisclosed to avoid unsupported or speculative manufacturer information.

All computational experiments were performed on a GPU workstation equipped with a single NVIDIA RTX PRO 6000 Blackwell Server Edition GPU (NVIDIA Corporation, Santa Clara, CA, USA). The software environment included Python (Version 3.12.13), PyTorch (Version 2.11.0+cu128), TorchVision (Version 0.26.0+cu128), timm (Version 1.0.27), NumPy (Version 2.0.2), pandas (Version 2.2.2), scikit-learn (Version 1.6.1), Matplotlib (Version 3.10.0), Pillow (Version 11.3.0), and CUDA Toolkit (Version 12.8; NVIDIA Corporation, Santa Clara, CA, USA). Mixed-precision GPU training was enabled where applicable.

### 3.1. Dataset

#### 3.1.1. Image Acquisition

In this study, a proprietary electroluminescence (EL) image dataset consisting of crystalline silicon photovoltaic cells was utilized. The dataset was collected from a single industrial inspection facility under controlled acquisition conditions. Consistent with previously established procedures [9], near-infrared EL images were captured using a silicon charge-coupled device (CCD) camera positioned at a fixed working distance from the photovoltaic modules. During image acquisition, the modules were operated under forward electrical bias at their short-circuit current level inside a darkroom environment designed to minimize ambient infrared interference. After acquisition, the full module images were segmented into individual solar cell regions through grid localization procedures. These isolated cell images were then used as the fundamental classification units throughout the benchmark. Since the original EL images were monochromatic, each grayscale image was replicated across three color channels prior to training. This preprocessing step enabled the direct utilization of ImageNet-pretrained architectures without modifying the first convolutional layers of the models, thereby preserving the pretrained feature representations. During training, all images were additionally normalized using ImageNet statistics. To ensure compatibility across all architectures evaluated in Section 3.4, every image was resized to a spatial resolution of 224 × 224 pixels. The final benchmark dataset contains 20,000 unique single-cell EL images, including 6000 monocrystalline and 14,000 polycrystalline samples. The detailed class-level dataset composition is summarized in Table 1, and the task-specific sample counts and fold distributions are subsequently reported in Table 2.

**Figure 1:**
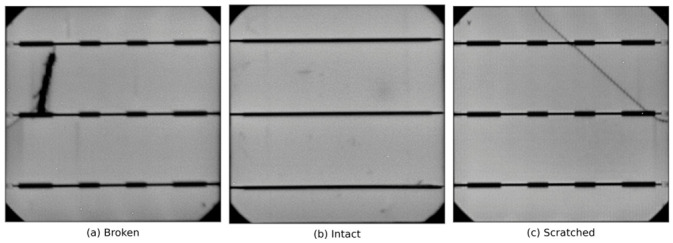
Representative EL images corresponding to the three monocrystalline defect categories: (**a**) broken, (**b**) intact and (**c**) scratched. All samples are presented as single-channel grayscale images at their native cell resolution.

The image acquisition process was performed by the industrial data provider using an electroluminescence inspection setup consisting of a silicon charge-coupled device camera, a darkroom acquisition environment, and a forward-bias electrical excitation unit. The authors did not directly operate the acquisition equipment, and the manufacturer/model information of the original camera, power supply, and darkroom system was not disclosed by the industrial partner under the data-sharing agreement. Therefore, these acquisition-device details are not reported to avoid unsupported or speculative manufacturer information. Representative monocrystalline electroluminescence examples are shown in Figure 1.

**Figure 2:**
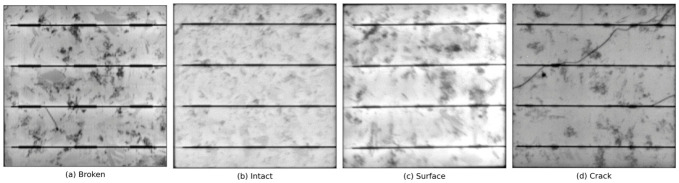
Representative EL images corresponding to the four polycrystalline defect categories: (**a**) broken, (**b**) intact, (**c**) surface-level degradation, and (**d**) crack.

Figure 2 presents representative electroluminescence images of the four polycrystalline photovoltaic cell categories considered in this study: (a) broken, characterized by severe structural damage, extensive fracture regions, and large electrically inactive dark areas indicating substantial disruption of current transport; (b) intact, exhibiting a relatively uniform EL emission pattern without visible cracks, inactive regions, or degradation signatures; (c) surface-level degradation, showing diffuse and spatially distributed darkening with low contrast and without clearly defined fracture boundaries; and (d) crack, identified by narrow dark line-like features corresponding to microcracks within the silicon structure.

#### 3.1.2. Panel Technologies and Defect Taxonomy

The dataset contains two crystalline silicon photovoltaic technologies: monocrystalline and polycrystalline solar cells. Separate label taxonomies were used because these materials exhibit different structural patterns in EL images. Monocrystalline cells have a relatively homogeneous crystal structure; therefore, linear mechanical defects such as scratches and severe broken regions are visually prominent. Polycrystalline cells have a heterogeneous grain structure and frequently show irregular low-contrast regions, which motivated an additional surface-level degradation class. The final monocrystalline taxonomy contains intact, scratched, and broken cells, while the polycrystalline taxonomy contains intact, cracked, broken, and surface-level degradation classes.

For clarity, the defect categories were interpreted as follows. Intact cells show largely uniform EL brightness without visually dominant fractures or inactive areas. Broken cells contain severe structural interruption or electrically inactive regions that appear as large dark areas, often reflecting handling, cutting, soldering, or mechanical stress. Scratched monocrystalline cells show thin or elongated surface marks that locally disturb the EL pattern without necessarily forming a through-cell crack. Cracked polycrystalline cells show narrow dark lines or branching fracture-like structures associated with mechanical or thermo-mechanical stress. Surface-level degradation refers to diffuse, mottled, or low-contrast darkening patterns on polycrystalline cells that are visually different from sharp crack lines; such regions may be associated with superficial abrasion, contamination, local degradation, or non-uniform surface response. These definitions were added to make the physical interpretation of Figure 2 explicit and to reduce ambiguity for readers unfamiliar with EL morphology.

#### 3.1.3. Annotation and Quality Control

All EL images included in the dataset were annotated by a domain expert experienced in photovoltaic (PV) inspection. To improve annotation reliability and reduce potential labeling errors, all assigned labels were independently reviewed by a second annotator. Whenever disagreements occurred, both annotators jointly re-evaluated the samples using the original full-module electroluminescence images as references. Final labels were determined through consensus between the annotators. Samples that remained ambiguous after the review process were excluded from the dataset in order to preserve label consistency and overall dataset quality. The final dataset was constructed with a strictly balanced class distribution within each panel category. For monocrystalline cells, the three classes, intact, scratched, and broken, each contain an equal number of samples. Similarly, for polycrystalline cells, the four categories intact, cracked, broken, and surface-level degradation are also equally represented within the dataset. The final dataset was constructed with a strictly balanced class distribution within each panel category. Table 1 summarizes the complete dataset composition according to panel type and defect category.

**Table 1:** Dataset composition according to panel type and defect category. Class distributions are balanced within each panel group.

Panel Type	Class	Count	Share Within Panel Type (%)
Monocrystalline	Intact	2000	33.3
Monocrystalline	Scratched	2000	33.3
Monocrystalline	Broken	2000	33.3
Polycrystalline	Intact	3500	25.0
Polycrystalline	Cracked	3500	25.0
Polycrystalline	Broken	3500	25.0
Polycrystalline	Surface-level	3500	25.0

For all classification tasks, a stratified five-fold cross-validation strategy was employed. In each fold, 20% of the data was allocated to the test subset, while the remaining 80% was further divided into training (85%) and validation (15%) partitions. Since the same pool of 20,000 unique EL cell images was reused across different tasks with varying label granularities, the total number of samples differs between tasks. Table 2 presents the dataset size and fold distribution used for each experimental task.

**Table 2:** Dataset sizes and stratified five-fold split configuration for each task. Values indicate the number of samples per fold.

Task	Classes	Total Images	Train/Fold	Val/fold	Test/Fold
T1 Type	2	16,027	10,897–10,898	1924	3205–3206
T2 Mono-Binary	2	4000	2720	480	800
T3 Mono-Detail	3	6000	4080	720	1200
T4 Poly-Binary	2	7000	4760	840	1400
T5 Poly-Detail	4	14,000	9520	1680	2800

#### 3.1.4. Class Balance and Binary Task Construction

At the most detailed labeling level, the dataset maintains perfectly balanced class distributions (Table 1). Each monocrystalline defect category contains 2000 cell images, whereas each polycrystalline defect category includes 3500 samples. For the binary classification tasks, all defective subclasses belonging to the same panel type were merged into a single defective category. To preserve strict class balance in these binary settings, the combined defective group was deterministically downsampled using a fixed random seed until it matched the number of intact samples. This procedure ensured identical class distributions across all folds and eliminated variability caused by random sampling differences. As a result, the binary monocrystalline task consists of 4000 images equally divided between intact and defective classes, while the binary polycrystalline task contains 7000 balanced samples. For the primary material classification task, a subset of images was selected from the full repository of 20,000 EL cell images. Sampling was terminated once the two material categories reached nearly identical representation levels, resulting in 8013 monocrystalline and 8014 polycrystalline samples respectively. Maintaining uniform class distributions across all tasks was considered essential for preventing biased performance estimation. Since photovoltaic datasets collected in real-world environments are naturally dominated by functional samples, models can artificially achieve high scores by favoring majority classes. By employing balanced datasets together with macro-averaged F1 evaluation, the proposed framework minimizes this methodological issue and ensures that all classes contribute equally to the final performance assessment.

#### 3.1.5. Availability and Reproducibility

The dataset used in this study is a proprietary collection specifically assembled for this benchmark and does not belong to publicly available EL datasets such as ELPV. To improve reproducibility despite this access constraint, the authors will release the exact train, validation, and test fold index files for every task, the deterministic downsampling seeds used to construct the binary tasks, the training configuration files, the model initialization details, training logs, and figure-generation scripts. During peer review, an anonymized archive containing these experimental artifacts can be shared with reviewers upon request. The raw grayscale PNG EL images are not publicly redistributed because of institutional and industrial data-sharing restrictions; however, access may be requested from the corresponding author under the applicable data-sharing protocol. This statement is intended to distinguish reproducible experimental configuration from unrestricted public image availability.

### 3.2. Task Hierarchy

Five classification tasks were defined to evaluate model behavior under progressively increasing defect complexity. Task 1 separates monocrystalline from polycrystalline cells and mainly tests whether models capture global grain-structure differences. Tasks 2 and 4 collapse all defects into binary intact/defective labels for mono- and polycrystalline subsets, reflecting common industrial screening use cases. Tasks 3 and 5 retain fine-grained defect labels and therefore test whether models can separate visually similar morphologies rather than merely detecting abnormality. The polycrystalline four-class task is expected to be the most difficult because surface-level degradation, cracks, and broken regions can overlap in texture, darkness, and spatial extent. By varying label granularity while holding image preprocessing and training constant, the benchmark isolates how architectural design affects progressively harder EL recognition scenarios.

### 3.3. Preprocessing and Augmentation

Before training, pixel intensities of all EL images were scaled to the 0–1 range and normalized using ImageNet statistics [30], allowing all ImageNet-pretrained backbones to be reused without modifying their first layers. Because EL defect cues can be subtle and localized, augmentation was deliberately conservative. Horizontal and vertical flips were allowed because single-cell EL images do not have a semantically fixed orientation, and brightness/contrast jitter was restricted to ±5% to avoid erasing low-contrast defect patterns. MixUp [31] was applied with α = 0.1 and 50% probability as a mild regularizer, and label smoothing [32] with ε = 0.1 was used to reduce overconfident predictions. Stronger policies such as CutMix or aggressive RandAugment were avoided because they can obscure cracks, scratches, and surface-level patterns that occupy only a small region of the cell.

### 3.4. Model Configurations

All neural network backbones were initialized using publicly available ImageNet-pretrained weights. For each task, the original classification layer was replaced with a task-specific linear head matching the number of target classes. The model set was intentionally designed to cover complementary deployment and representation regimes: MobileNetV3-Large tests a mobile-efficient CNN, DenseNet121 tests a compact classical CNN, ConvNeXt-T tests a modern hierarchical CNN, Swin-T tests a hierarchical transformer, and ViT-B/16 and DeiT-B/16 test base-scale transformer variants. This selection allows the benchmark to evaluate whether transformer-scale capacity is necessary for EL-based PV defect classification, or whether a modern convolutional design can provide a better balance between accuracy and complexity. The backbone architectures evaluated in this study, together with their architectural family, parameter count, and input resolution, are summarized in Table 3.

### 3.5. Training Protocol

All models were optimized using AdamW [33]. The initial learning rate was set to 1 × 10^−4^ for convolution-based architectures and 5 × 10^−5^ for transformer-based architectures to account for the greater sensitivity of transformer fine-tuning. Weight decay was fixed at 0.05 across all experiments, and cosine annealing was used over a maximum of 30 epochs. A batch size of 32 was used for all models; for ViT-B/16 and DeiT-B/16, gradient accumulation maintained the same effective batch size under memory constraints. Cross-entropy loss with label smoothing was used for all tasks, and mixed-precision training was enabled to reduce memory usage. Early stopping with patience of eight epochs was applied based on validation macro-F1 to reduce overfitting, especially in smaller tasks. Temperature scaling [34] was fitted on each validation split for probability calibration, although it did not affect the reported macro-F1 values. In total, the benchmark required 150 independent training runs corresponding to 6 backbones, 5 tasks, and 5 folds. All experiments were executed on the GPU workstation described at the beginning of Section 3, and the full training process required 755.9 min, corresponding to approximately 12.6 h of total wall-clock time.

All experiments were evaluated using stratified five-fold cross-validation implemented in scikit-learn [35] with fixed random seeds. In each fold, 20% of the data was reserved for testing, while the remaining 80% was split into training (85%) and validation (15%) subsets, yielding an overall distribution of 68% training, 12% validation, and 20% testing per-fold. The same fold definitions were reused across all architectures within each task to ensure fair paired comparison. Accuracy, macro-F1, weighted F1, macro precision, and macro recall were reported as mean ± standard deviation across folds, with macro-F1 selected as the primary metric because it gives equal weight to each class.

A key validity consideration is the distinction between image-level and module-level evaluation. The current benchmark uses image-level stratified cross-validation. This guarantees class balance and paired model comparison, but it does not strictly prevent adjacent cells from the same PV module from appearing in different folds. If module-specific acquisition patterns are learned, absolute performance values may therefore be slightly optimistic. For this reason, the results should be interpreted as controlled image-level cross-validation rather than as a fully module-independent generalization estimate. A stricter follow-up protocol should use module-level grouped cross-validation, where all cells originating from the same physical module are assigned to only one fold. This limitation is explicitly revisited in the Discussion, and the released fold metadata should be extended with module identifiers whenever institutional data-sharing rules permit.

**Figure 3:**
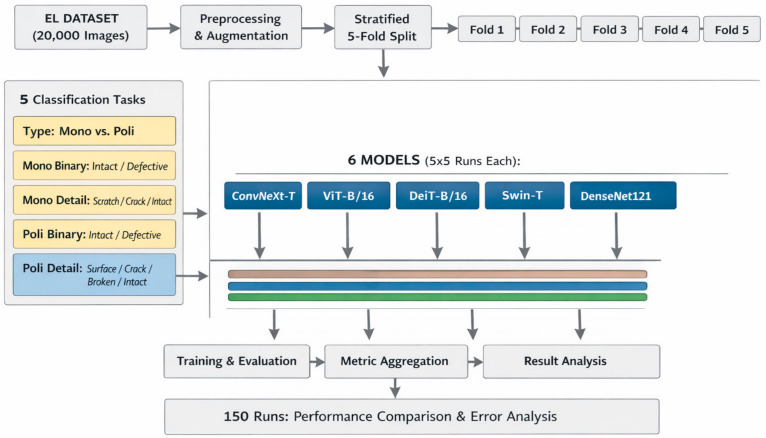
Benchmark workflow. The yellow blocks indicate the five classification tasks, the blue blocks indicate the six evaluated backbone architectures, and the arrows show the flow from dataset preparation and stratified five-fold splitting to model training, metric aggregation, and error analysis.

For statistical comparison, paired Wilcoxon signed-rank tests were applied across folds for each ConvNeXt-T versus comparator pair. Because the number of folds is five, the smallest attainable two-sided *p*-value is 0.0625; therefore, *p*-values were interpreted together with directional consistency across folds, and Holm–Bonferroni correction was applied when multiple comparisons were assessed within the same task. The overall benchmark workflow, including dataset preparation, stratified five-fold splitting, task definitions, model training, and evaluation stages, is summarized in Figure 3.

For each of the five defined tasks, the corresponding subset of the 20,000-image EL dataset (Section 3.1) is divided into five stratified folds. Each fold is then used to independently fine-tune all six ImageNet-pretrained backbones under the unified training protocol described in Section 3.5, with 30 epochs of training per-fold. All reported results in Section 4 are obtained from independently trained single-task models, where each architecture is optimized end-to-end on the respective task-specific dataset. The mono-to-poly and binary-to-detailed progression shown in the figure reflects the hierarchical label structure defined in Section 3.1.2, rather than a sequential inference pipeline.

## 4. Results

This section evaluates six deep learning backbones across five electroluminescence-based photovoltaic defect classification tasks using a strictly controlled stratified five-fold cross-validation setup. Performance is mainly measured with macro-averaged F1-score, supported by precision, recall, and weighted F1 for a more detailed analysis. The results are presented as overall performance followed by task-level comparisons, highlighting how model behavior changes with increasing task difficulty. Additional analyses cover parameter efficiency, class-wise errors, fold stability, and statistical significance to ensure a fair and reproducible comparison across different architectural families.

### 4.1. Overall Performance

ConvNeXt-T achieves the highest aggregate score (93.12%), followed closely by ViT-B/16 (92.81%) and DeiT-B/16 (92.39%). Swin-T ranks fourth with a mean score of 90.21%, while DenseNet121 (86.07%) and MobileNetV3-Large (77.42%) demonstrate considerably lower performance. These results reveal a clear hierarchy among the evaluated models, where modern convolutional and transformer-based architectures consistently outperform lightweight convolutional networks. Notably, although the performance gap between ConvNeXt-T and the base transformer models is relatively small at the overall level, it becomes more pronounced when individual tasks are considered. A more detailed examination shows that performance varies across tasks. ConvNeXt-T achieves the best results in three out of five tasks (Mono-Binary, Poly-Binary, and Poly-Detail), whereas ViT-B/16 and DeiT-B/16 each attain the highest performance in one task (Mono-Detail and Type, respectively). For the simpler tasks Type, Mono-Binary, and Mono-Detail—all leading models exceed 98% macro F1-score, indicating that these tasks are largely saturated and therefore less discriminative in distinguishing between architectures. In contrast, the polycrystalline-related tasks introduce a clearer separation among models. In particular, performance differences become more evident in the Poly-Detail task, where architectural capacity plays a more decisive role. Lightweight models exhibit the most significant performance degradation in these challenging settings, highlighting their limited ability to capture complex defect structures. The mean macro-F1 scores obtained across the five-fold cross-validation experiments for all evaluated backbones and tasks are summarized in Table 4.

### 4.2. Per-Task Analysis

**Figure 4:**
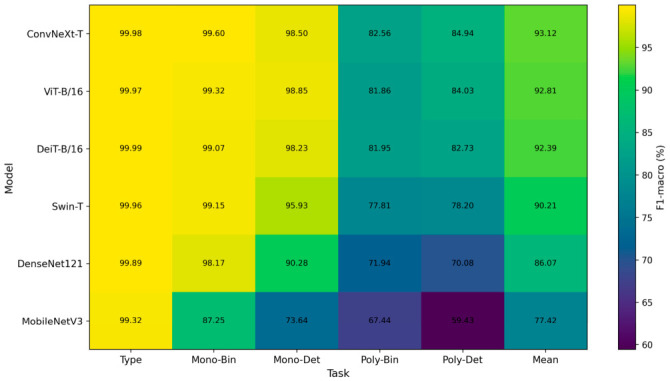
Macro-F1 heatmap across models and tasks. Color intensity represents macro-F1 score, where darker purple/blue tones indicate lower performance and brighter green/yellow tones indicate higher performance. The rightmost column reports the mean performance of each model across all five tasks.

Performance varies considerably across tasks, revealing clear differences in how architectures behave as task complexity increases. The primary material classification task is effectively saturated, with all models exceeding 99.3% macro F1-score. DeiT-B/16 achieves the highest performance (99.99 ± 0.01%), although the differences between models are negligible. This near-perfect performance across all architectures suggests that the task is dominated by easily separable global texture cues, particularly the distinction between uniform monocrystalline structures and irregular polycrystalline grain patterns. As a result, it offers limited discriminative power for comparing model capabilities. The monocrystalline defect detection tasks (binary and multi-class) remain relatively straightforward for the strongest models, all of which maintain performance above 98% macro F1-score. Nevertheless, these tasks begin to expose the limitations of lightweight architectures. MobileNetV3-Large, in particular, experiences a noticeable drop in performance on the binary task (87.25 ± 3.76%), accompanied by increased variance across folds. This indicates reduced robustness and higher sensitivity to data partitioning. The polycrystalline-related tasks constitute the most challenging part of the benchmark and provide the clearest separation between architectures. In the Poly-Binary task, ConvNeXt-T achieves the highest performance (82.56 ± 1.35%), slightly outperforming ViT-B/16 (81.86 ± 4.17%). Although the mean difference is small, the substantially higher variance of ViT-B/16 suggests less stable behavior. DeiT-B/16 shows slightly lower mean performance (81.95 ± 1.70%) but demonstrates more consistent results across folds. This separation becomes even more evident in the Poly-Detail task. ConvNeXt-T again leads with 84.94 ± 0.45%, followed by ViT-B/16 (84.03 ± 0.21%) and DeiT-B/16 (82.73 ± 0.50%). Swin-T shows a further decline in performance (78.20 ± 1.31%), while DenseNet121 (70.08 ± 1.19%) and MobileNetV3-Large (59.43 ± 1.71%) lag significantly behind. Overall, these findings underline the importance of architectural capacity and design choices in capturing fine-grained defect patterns under complex visual conditions behind. Overall, these findings underline the importance of architectural capacity and design choices in capturing fine-grained defect patterns under complex visual conditions. To visualize the task-wise performance distribution across all evaluated backbones, the macro-F1 heatmap is presented in Figure 4.

**Figure 5:**
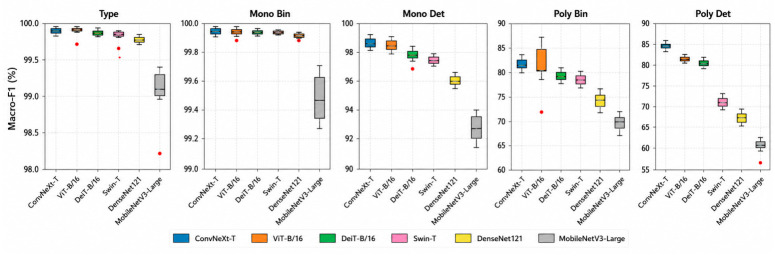
Per-fold macro-F1 distributions across the five tasks. Each panel corresponds to one classification task, and each box summarizes the five cross-validation fold values for a given backbone. The colored boxes denote the evaluated backbone architectures in the same left-to-right order within each panel: ConvNeXt-T, ViT-B/16, DeiT-B/16, Swin-T, DenseNet121, and MobileNetV3-Large. The central line inside each box indicates the median, the box limits indicate the interquartile range, and the whiskers show the remaining fold-wise variation. Red markers indicate outlier fold values. Taller boxes indicate greater fold sensitivity, whereas compact boxes indicate more stable performance across folds. The larger ViT-B/16 box on the Poly-Binary task is mainly driven by one outlier fold.

To further assess model robustness across cross-validation folds, the per-fold macro-F1 distributions are shown in Figure 5.

ConvNeXt-T consistently shows tightly clustered score distributions, indicating stable and reliable performance across different data splits. In contrast, ViT-B/16 exhibits a noticeably broader distribution on the Poly-Binary task, largely driven by a single low performing fold. This leads to an increased standard deviation and suggests greater sensitivity to variations in the training data composition. Moreover, only a limited overlap is observed between ConvNeXt-T and the other architectures on the Poly-Detail task. This clear separation further highlights the advantage of ConvNeXt-T on the most challenging classification scenario within the benchmark.

### 4.3. Parameter Efficiency

**Figure 6:**
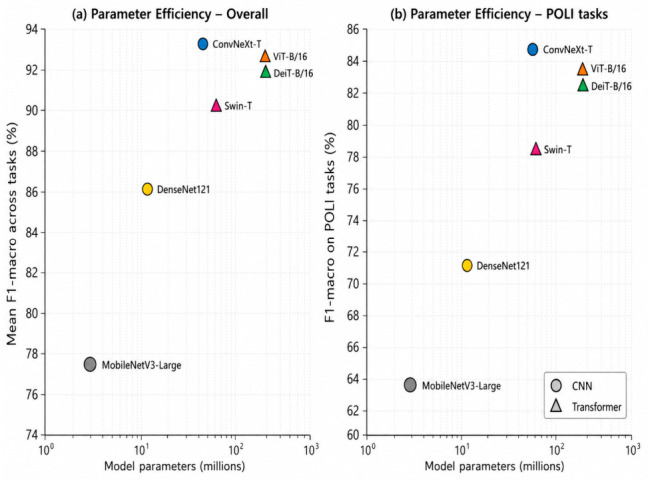
Mean F1 macro vs. parameter count (log scale). ConvNeXt-T achieves the highest mean F1 macro with roughly one third of the parameters of the two base-size vision transformers. The Pareto front includes ConvNeXt-T, Swin-T and DenseNet121.

A common assumption is that the larger parameter counts of vision transformers should translate into superior fine-grained visual recognition. The present results do not support this assumption under the current EL benchmark. ConvNeXt-T achieves the highest mean macro-F1 (93.12%) with 27.82 million parameters, whereas ViT-B/16 and DeiT-B/16 contain approximately 85.80 million parameters and achieve lower mean scores of 92.81% and 92.39%, respectively. This indicates that architectural bias and feature hierarchy, not only parameter scale, are important for EL defect classification. ConvNeXt-T combines convolutional locality with transformer-inspired design choices such as larger effective receptive fields, inverted bottlenecks, and normalization choices, which may help it capture both localized cracks and broader surface-level texture variation. Figure 6 summarizes the accuracy–parameter relationship, where ConvNeXt-T lies near the Pareto frontier for this benchmark. ConvNeXt-T also provides the most favorable accuracy–efficiency trade-off among the high-performing models, combining the highest mean macro-F1 with substantially lower batch-1 inference latency and peak GPU memory than the base ViT/DeiT backbones under the NVIDIA RTX PRO 6000 Blackwell Server Edition profiling setup reported in Table 5.

Latency measurements were performed using a batch size of 1 and an input resolution of 224 × 224 pixels on an NVIDIA RTX PRO 6000 Blackwell Server Edition GPU under FP32 inference. Reported latency values represent the mean ± standard deviation obtained from 100 timed forward passes after a warm-up phase, excluding image loading and preprocessing operations. Peak memory corresponds to the maximum GPU memory allocated during inference. FLOPs are approximate per-image estimates derived from standard ImageNet implementations and are included as architecture-level indicators of computational complexity. The results reveal notable differences in the balance between predictive performance and computational efficiency across the evaluated architectures. MobileNetV3-Large exhibits the lowest computational burden, achieving the smallest parameter count, memory footprint, and inference latency. However, this efficiency comes at the cost of a substantial reduction in classification performance, yielding the lowest macro-F1 score among all evaluated models. This finding suggests that highly compact architectures may struggle to capture the subtle texture variations and defect patterns characteristic of polycrystalline photovoltaic cell electroluminescence images. Among the higher-performing models, ConvNeXt-T demonstrates the most favorable overall trade-off between accuracy and deployment efficiency. Despite having a parameter count comparable to Swin-T, ConvNeXt-T achieves a higher mean macro-F1 score while maintaining lower inference latency and slightly reduced memory requirements. Furthermore, although ViT-B/16 and DeiT-B/16 attain competitive classification performance, both transformer-based architectures require more than three times the parameter storage and approximately four times the computational complexity of ConvNeXt-T, resulting in significantly higher latency and memory consumption. From a deployment perspective, ConvNeXt-T achieves the highest mean macro-F1 score (93.12%) while maintaining moderate computational requirements (4.47 GFLOPs), a compact FP32 memory footprint (106.1 MB), and an inference latency of only 5.3 ms. These characteristics indicate that ConvNeXt-T provides the most balanced operating point among the evaluated backbones, making it particularly suitable for real-time photovoltaic defect inspection systems where both classification reliability and computational efficiency are critical considerations.

### 4.4. Precision–Recall Asymmetry and Minority Class Failure

**Figure 7:**
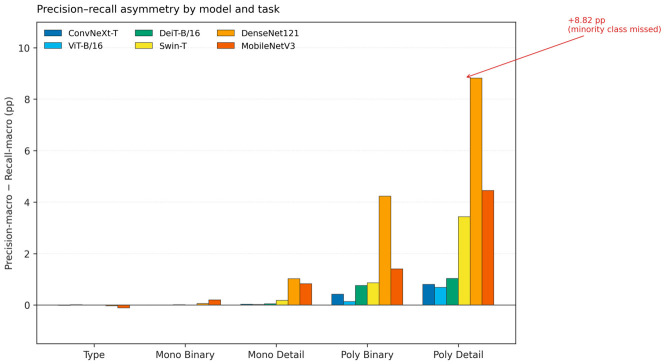
Precision-macro minus recall-macro, in percentage points, for each model and task. The largest asymmetry is DenseNet121 on Poly-Detail (+8.82 pp), which is a minority-class failure signal rather than an overall-accuracy problem.

The macro F1 score provides an aggregated view of overall performance, yet it often conceals where residual classification errors originate particularly whether they are concentrated in dominant classes or rare defect morphologies. Examining the gap between macro precision and macro recall offers a more transparent view of this error distribution without requiring additional analytical structures. A positive gap, where macro precision exceeds macro recall, reflects a conservative prediction behavior--which systematically fails to detect minority instances. In contrast, a negative gap indicates an over-predictive tendency toward these rare occurrences, suggesting a more aggressive decision boundary. Figure 7 illustrates this divergence across all architecture and task combinations, highlighting how precision–recall asymmetry varies depending on the model configuration.

Across the three fundamental classification tasks, all evaluated architectures exhibit highly consistent performance, with metric differentials remaining strictly below one percentage point. This near-equivalence between macro precision and macro recall indicates that the aggregate macro F1 score serves as a reliable and representative summary of overall model behavior in these settings. In contrast, the polycrystalline evaluation regime reveals a more heterogeneous pattern. Architectures such as ConvNeXt-T, ViT-B/16, and DeiT-B/16 preserve relatively balanced behavior, with precision recall discrepancies remaining below approximately 1.1 percentage points. This suggests that their residual errors are evenly distributed across classes, without pronounced bias toward or against minority defect categories. However, Swin-T exhibits a modest increase in asymmetry (+3.43 pp on Poly-Detail), indicating a slight drift toward uneven error allocation. This trend becomes more pronounced in smaller convolutional backbones. DenseNet121 demonstrates the most substantial imbalance across the entire benchmark, with +8.82 pp on Poly-Detail and +4.23 pp on Poly-Binary. MobileNetV3-Large similarly shows elevated divergence (+4.45 pp on Poly-Detail). In both DenseNet121 and MobileNetV3-Large, macro precision is consistently higher than macro recall for instance, in DenseNet121 on Poly-Detail (Precision-M: 79.27%, Recall-M: 70.45%). This pattern is indicative of a conservative failure mode in which the models under-detect rare defect classes, leading to systematic omission rather than over-prediction. Notably, this behavior dominates the residual error structure of lightweight convolutional architectures in polycrystalline settings and remains obscured when relying solely on aggregate metrics such as accuracy or macro F1.

### 4.5. Per-Class Confusion Analysis on Poly-Detail

**Figure 8:**
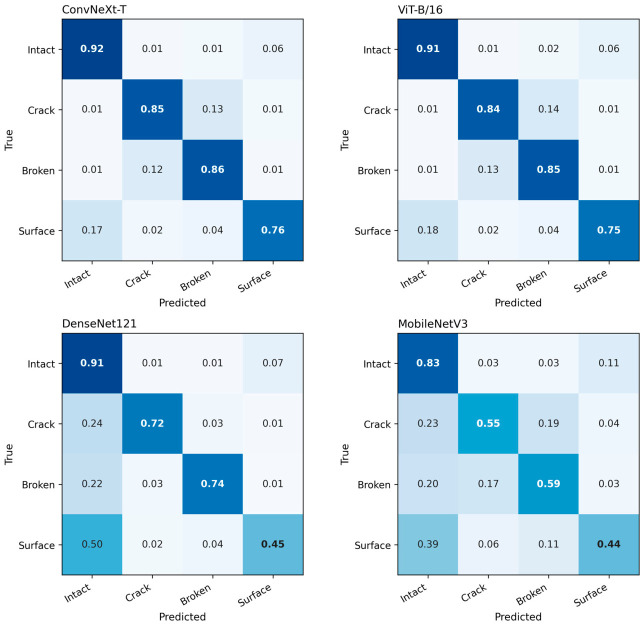
Row-normalized confusion matrices on Poly-Detail, aggregated across all five folds. The four backbones span the full performance range from ConvNeXt-T (**top-left**) to MobileNetV3-Large (**bottom-right**). Off-diagonal mass concentrates on two class pairs: intact–surface-level and crack–broken.

**Figure 9:**
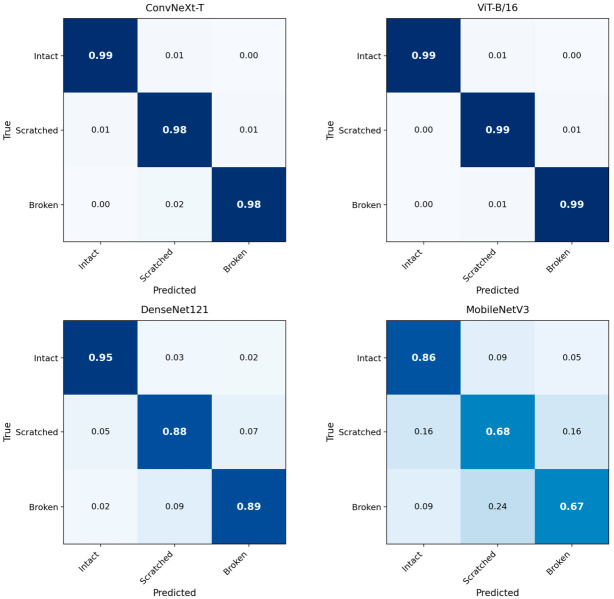
Row-normalized confusion matrices on Mono-Detail, aggregated across all five folds. The four backbones span the full performance range from ConvNeXt-T (**top-left**) to MobileNetV3-Large (**bottom-right**). Remaining errors concentrate on the scratched–broken pair; the intact class is essentially separable for all backbones except MobileNetV3-Large.

**Figure 10:**
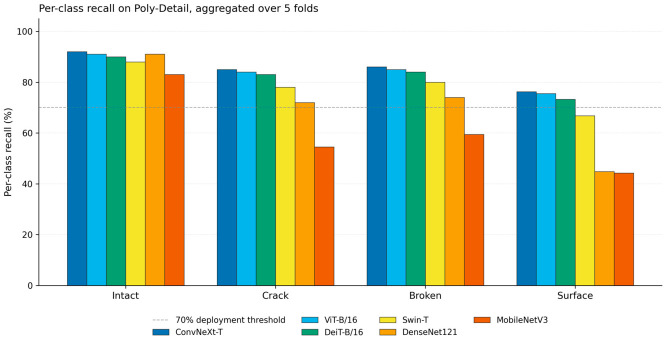
Per-class recall on Poly-Detail across the six backbones, aggregated over all five folds. The dashed line at 70% marks a conservative deployment threshold. Small backbones fall below this line on the two rarer defect classes, confirming the minority class failure signal of Section 4.4.

The precision–recall variance introduced in Section 4.4 provides a compact, scalar-level indication that lightweight models struggle disproportionately with difficult defect categories in the fine-grained polycrystalline setting. However, this aggregated view does not reveal where these errors originate in terms of specific class-to-class confusions. To move beyond this limitation, we aggregate the held-out test-fold confusion matrices for four representative architectures: ConvNeXt-T, ViT-B/16, DenseNet121, and MobileNetV3-Large. Figure 8 presents the row-normalized confusion matrices, emphasizing recall behavior and making it possible to directly observe how samples from each class are redistributed across incorrect predictions. To move beyond this limitation, we aggregate the held-out test-fold confusion matrices for four representative architectures: ConvNeXt-T, ViT-B/16, DenseNet121, and MobileNetV3-Large. Figure 8 presents the row-normalized confusion matrices for the Poly-Detail task, emphasizing recall behavior and making it possible to directly observe how samples from each class are redistributed across incorrect predictions. The corresponding monocrystalline detailed-task confusion matrices are shown in Figure 9. Complementing these confusion-matrix results, Figure 10 and Table 6 provide a detailed breakdown of per-class recall scores for all models, enabling a more granular comparison of failure patterns at the individual class level.

Two off-diagonal misclassification patterns stand out clearly in the Poly-Detail evaluation. The first concerns the systematic confusion between intact and superficial classes. In polycrystalline modules, diffuse superficial degradation produces subtle contrast reduction and mild darkening in electroluminescence images. At the 224 × 224 input resolution, these visual cues can closely resemble the texture of healthy cells, making the distinction inherently challenging. As a result, all architectures exhibit some degree of overlap between these two states. However, ConvNeXt-T demonstrates a noticeably stronger separation, maintaining consistently higher recall for superficial defects. In contrast, DenseNet121 and MobileNetV3-Large misclassify a significant portion of genuinely degraded samples as intact, which directly explains the pronounced recall collapse observed for the superficial class in these lighter convolutional baselines.

The second dominant pattern involves confusion between cracked and broken states. These two categories lie on a natural continuum of structural degradation, where severe cracks often progress into complete cell failure. Consequently, their electroluminescence signatures share strong morphological similarities, particularly in regions surrounding fracture propagation. While the top-performing backbones still manage to separate these classes with per-class recall values above 80%, DenseNet121 and MobileNetV3-Large show a clear bias toward one side of this pair, producing an asymmetric off-diagonal block that is especially visible in the lower region of Figure 8. This imbalance is also reflected in the widened crack–broken gap observed in the per-class recall curves in Figure 10. Importantly, this specific confusion structure is not observable in the binary Poly evaluation, where merging crack and broken into a single “defective” category effectively hides the underlying ambiguity. This highlights why fine-grained evaluation is essential in photovoltaic defect analysis: aggregate binary metrics can mask structurally meaningful failure modes that only emerge at higher label resolution.

The confusion-matrix and per-class recall analyses provide a dataset-level explanation of the observed performance gaps by identifying the main missed-defect categories and class-pair confusions. These findings are complemented by the instance-level Grad-CAM and attention visualizations reported in Section 4.8.

### 4.6. Cross-Validation Stability

**Figure 11:**
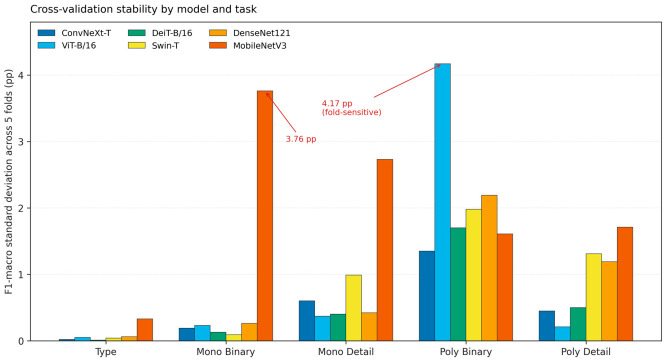
Standard deviation of macro F1 across five folds per model and task. Two notable outliers are observed: ViT-B/16 on Poly-Binary (σ = 4.17 pp), driven by a single degraded fold where one class effectively collapsed, and MobileNetV3-Large on Mono-Binary (σ = 3.76 pp).

Mean performance alone is insufficient for assessing suitability in a deployed screening system. Equally important is the stability of the model across different data splits, as fold-to-fold variance reflects how dependent the reported performance is on a particular partitioning of the dataset. Figure 11 reports the standard deviation of macro F1 scores across the five cross-validation folds for each model task pair.

Two distinct instability patterns emerge. The first concerns ViT-B/16 on the Poly-Binary task, where the standard deviation reaches 4.17 percentage points, compared to much lower values for ConvNeXt-T (1.35 pp) and DeiT-B/16 (1.70 pp) under the same setting. A closer inspection of per-fold results reveals that four folds remain highly consistent, while a single fold exhibits a substantial performance drop, which disproportionately inflates the overall variance. This behavior is consistent with the known sensitivity of baseline vision transformers to dataset composition, particularly in scenarios lacking strong inductive biases such as locality or hierarchical feature modeling. In such cases, limited test set size and distributional shifts across folds can lead to pronounced instability, as pure attention-based architectures lack mechanisms to inherently regularize spatial consistency. The second anomaly appears in MobileNetV3-Large on the Mono-Binary task, where σ = 3.76 pp coincides with a relatively low mean performance (87.25%). Unlike the previous case, this instability is not driven by a single pathological fold but rather by inconsistent behavior across multiple splits. The model fails in different ways depending on the fold, despite the task being comparatively simple. This lack of robustness across nominally homogeneous evaluation settings represents a critical concern for deployment, as it indicates that performance is not only suboptimal but also unpredictable across data partitions.

### 4.7. Statistical Tests

**Figure 12:**
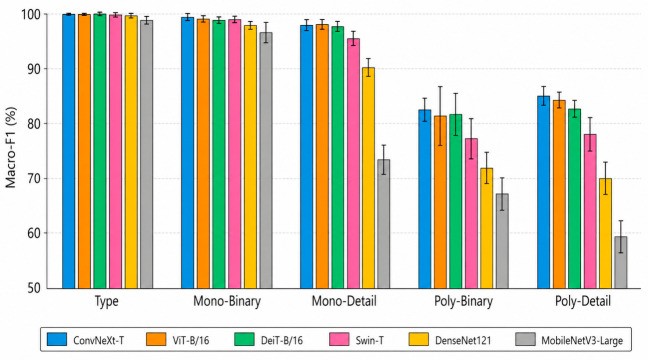
Task-difficulty hierarchy with per-fold standard deviations. Bar heights indicate mean macro-F1 scores, and error bars indicate fold-level standard deviations. Colors denote the evaluated backbone architectures: ConvNeXt-T, ViT-B/16, DeiT-B/16, Swin-T, DenseNet121, and MobileNetV3-Large. Three tasks are saturated or near-saturated (Type, Mono-Binary, and Mono-Detail), one is moderately difficult (Poly-Binary), and one is visually the most challenging (Poly-Detail), where architectural choice has the largest effect.

Paired Wilcoxon signed-rank tests are computed across folds for each ConvNeXt-T versus comparator model pair. On the Poly-Detail task, ConvNeXt-T consistently outperforms all alternative architectures ViT-B/16, DeiT-B/16, Swin-T, DenseNet121, and MobileNetV3-Large in all five folds, yielding perfect directional agreement (5/5). This results in a test statistic of W = 0 with a two-sided *p*-value of 0.0625, corresponding to the minimum achievable significance level for *n* = 5. Although the limited number of folds restricts statistical granularity, the absence of any directional reversals indicates a robust and stable performance advantage for ConvNeXt-T in this setting. For the Poly-Binary task, the pattern is less consistent. Directional agreement decreases to 2/5 against ViT-B/16 and 4/5 against DeiT-B/16. This aligns with the smaller absolute performance gaps reported in Table 4 and is further influenced by the fold-specific outlier observed in ViT-B/16. Together, these results suggest that ConvNeXt-T’s advantage persists on average but is less uniformly expressed across individual folds in the binary polycrystalline regime. Across the remaining three, easier tasks, none of the pairwise comparisons achieve statistical significance after Holm–Bonferroni correction at α = 0.10. This outcome is expected given the near-saturated performance range (98–100% macro F1), where all architectures perform at a similarly high level and fold-level differences do not exhibit consistent directional structure. The resulting task difficulty hierarchy, derived from the full set of statistical comparisons, is visualized in Figure 12.

### 4.8. Robustness, Explainability, and Ablation Scope

Additional robustness considerations are interpreted within the methodological scope of the available benchmark. The present study does not report noisy-label, limited-data, or cross-domain transfer results because these experiments would require additional controlled training runs, alternative labeled domains, or module-level metadata beyond the current benchmark package. Instead, robustness is evaluated conservatively through repeated five-fold training, fold-level variance, precision–recall asymmetry, and per-class recall on the most difficult Poly-Detail task. These analyses should be interpreted as within-dataset robustness evidence, not as proof of cross-domain or field-deployment robustness. To complement the quantitative error analysis, instance-level visual explanations were generated for representative Poly-Detail held-out test samples. Grad-CAM was used for the convolutional backbones ConvNeXt-T and DenseNet121, while attention-based visualization was examined for ViT-B/16. ConvNeXt-T generally produced more spatially focused activation maps around crack boundaries, branching fracture patterns, and diffuse low-contrast regions characteristic of surface-level degradation. DenseNet121 showed broader and less localized activation patterns, particularly in samples containing subtle surface-level mottling. ViT-B/16 captured broader contextual regions but sometimes distributed attention over visually less relevant background structures. These qualitative patterns support the quantitative findings in Section 4.5 and suggest that the hierarchical convolutional design of ConvNeXt-T is well aligned with the localized and multi-scale morphology of EL defects.

For ConvNeXt-T, the study provides an architecture-level explanation rather than a destructive component ablation. Because ConvNeXt-T is evaluated as a standard ImageNet-pretrained backbone, removing large-kernel depthwise convolutions, inverted bottlenecks, or LayerNorm would create non-standard, differently pretrained variants and would no longer preserve the controlled backbone-comparison design. The manuscript therefore interprets ConvNeXt-T through its known architectural biases and reports full component-level ablation as a future extension rather than presenting unsupported ablation claims.

## 5. Discussion

The main contribution of this study is a controlled benchmark rather than a new defect-classification architecture. This distinction is important for interpreting the novelty of the work. The value of the study lies in comparing representative CNN, transformer, hierarchical transformer, and lightweight backbones under the same dataset, preprocessing, augmentation, training schedule, and fold definitions. Under this controlled setting, ConvNeXt-T shows the strongest balance between macro-F1, fold stability, and parameter efficiency, particularly on the four-class polycrystalline task where defect morphologies are visually similar.

Compared with prior EL-based PV defect studies, direct numerical comparison remains difficult because datasets, class definitions, acquisition conditions, and evaluation protocols differ substantially. Many earlier studies use public ELPV subsets, binary or coarse labels, single train–test splits, or augmentation-specific protocols. The present benchmark adds value by explicitly reporting fold-to-fold variability across 150 independent runs and by separating mono- and polycrystalline recognition difficulty. Table 7 summarizes representative recent studies and highlights that repeated cross-validation and fine-grained polycrystalline analysis are still uncommon in this domain.

Several limitations should be considered. First, the dataset is proprietary and the raw EL images cannot be released publicly under the current data-sharing agreement. To partially mitigate this, fold indices, configuration files, deterministic seeds, and code artifacts should be released, and reviewer access to anonymized experimental artifacts should be provided when possible. Second, the present split strategy is image-level stratified cross-validation. Although this enables fair paired comparison across architectures, it does not fully eliminate the possibility that cells from the same module appear in different folds. A module-level grouped cross-validation protocol would provide a stricter estimate of module-independent generalization and should be implemented when reliable module identifiers can be distributed with the anonymized metadata.

Third, the benchmark focuses on supervised classification with balanced classes. Real industrial inspection pipelines often face noisy labels, natural class imbalance, camera/domain shifts, unseen module types, and varying image quality. The present revision therefore does not claim deployment robustness beyond the controlled image-level benchmark; instead, it identifies fold stability, per-class recall, measured single-GPU inference profiling, and saliency-based qualitative inspection as internal diagnostics, and specifies label-noise sensitivity, limited-data training, cross-domain testing, naturally imbalanced evaluation, and target edge-device profiling as the next required validation steps. Fourth, although batch-1 inference latency and peak GPU memory are reported on an NVIDIA RTX PRO 6000 Blackwell Server Edition GPU, these measurements should not be interpreted as universal deployment latency because hardware, precision mode, software stack, and preprocessing pipelines can substantially affect runtime behavior.

This study evaluated six deep learning architectures across five hierarchical photovoltaic defect classification tasks using electroluminescence imagery. Under a controlled image-level stratified five-fold protocol, 150 independent training runs were conducted with identical preprocessing, augmentation, optimization, and evaluation settings. ConvNeXt-T achieved the highest mean macro-F1 score (93.12%) and the strongest performance on the most challenging four-class polycrystalline task (84.94 ± 0.45% macro-F1), while using substantially fewer parameters than base ViT/DeiT models. The error analysis showed that smaller CNNs, especially DenseNet121 and MobileNetV3-Large, tend to under-detect difficult polycrystalline defect categories such as surface-level degradation and crack-related patterns. The added deployment profiling further indicates that ConvNeXt-T maintains this advantage with lower batch-1 latency and peak GPU memory than base ViT/DeiT models on the tested NVIDIA RTX PRO 6000 Blackwell Server Edition setup, while the qualitative Grad-CAM/attention analysis provides visual support for the localized defect sensitivity observed in the quantitative results. These findings support the use of modern hierarchical convolutional architectures as competitive candidates for EL-based PV inspection when accuracy, fold stability, parameter efficiency, and measured inference efficiency are considered together. At the same time, the conclusions remain bounded by the proprietary dataset and image-level cross-validation design. Future work should add module-level grouped validation, public- or reviewer-accessible metadata, noisy-label and cross-domain robustness tests, naturally imbalanced evaluation, larger-scale edge-device profiling, and broader fold-matched visual explanation analysis.

## 6. Conclusions

We evaluated six deep learning architectures across five hierarchical photovoltaic defect classification tasks using electroluminescence imagery. The benchmarking framework was based on stratified five-fold cross validation, resulting in 150 independent training runs under identical preprocessing pipelines, optimization settings, and loss functions. This design ensures that all comparisons are made under fully controlled experimental conditions. Among the evaluated models, ConvNeXt-T achieved the highest mean macro-F1 score (93.12%), and it was the best-performing model in three out of five tasks, despite using approximately one-third of the parameters of base-size ViT and DeiT architectures. In the most challenging setting, the four-class polycrystalline defect classification task, ConvNeXt-T reached 84.94 ± 0.45% macro-F1. In comparison, DenseNet121 and MobileNetV3-Large obtained substantially lower performance, with 70.08 ± 1.19% and 59.43 ± 1.71% macro-F1, respectively. Importantly, ConvNeXt-T maintained a consistent advantage across all five folds in this task against every competing model, indicating not only higher mean performance but also greater stability. A further analysis of precision recall behavior, derived from macro averaged metrics, reveals a systematic failure mode in smaller convolutional architectures. In particular, DenseNet121 exhibits an 8.82 percentage point gap between macro precision and macro recall in the polycrystalline setting, indicating a clear tendency toward under-detection of minority defect classes. This type of asymmetry is not observable from accuracy or macro-F1 alone, yet it represents a critical failure mode in practical inspection systems where missed defects are significantly more costly than false alarms. The results suggest that modern hierarchical convolutional networks such as ConvNeXt provide a more efficient and robust alternative to base scale vision transformers for electroluminescence-based photovoltaic inspection. Furthermore, the study highlights the importance of cross validated evaluation and per fold variance reporting as essential components of reliable benchmarking in this domain.

## Figures and Tables

**Table 3:** Backbone architectures evaluated in this study.

Model	Family	Parameters (M)	Input Resolution
ConvNeXt-T	Modern CNN (hierarchical)	27.82	224 × 224
Swin-T	Hierarchical transformer	27.52	224 × 224
DenseNet121	Classical CNN (dense)	6.96	224 × 224
MobileNetV3-Large	Mobile CNN	4.20	224 × 224
ViT-B/16	Plain transformer	85.80	224 × 224
DeiT-B/16	Distilled transformer	85.80	224 × 224

**Table 4:** Mean macro F1-score (%) across five-fold cross-validation for each model and task. Values are reported as mean ± standard deviation. The “Mean” column represents the average across all five tasks. Best-performing models per task are indicated in bold.

Model	Params (M)	Type	Mono-Bin	Mono-Det	Poly-Bin	Poly-Det	Mean
ConvNeXt-T	27.82	99.98 ± 0.02	**99.60 ± 0.19**	98.50 ± 0.60	**82.56 ± 1.35**	**84.94 ± 0.45**	**93.12**
ViT-B/16	85.80	99.97 ± 0.05	99.32 ± 0.23	**98.85 ± 0.37**	81.86 ± 4.17	84.03 ± 0.21	92.81
DeiT-B/16	85.80	**99.99 ± 0.01**	99.07 ± 0.13	98.23 ± 0.40	81.95 ± 1.70	82.73 ± 0.50	92.39
Swin-T	27.52	99.96 ± 0.04	99.15 ± 0.09	95.93 ± 0.99	77.81 ± 1.98	78.20 ± 1.31	90.21
DenseNet121	6.96	99.89 ± 0.06	98.17 ± 0.26	90.28 ± 0.42	71.94 ± 2.19	70.08 ± 1.19	86.07
MobileNetV3	4.20	99.32 ± 0.33	87.25 ± 3.76	73.64 ± 2.73	67.44 ± 1.61	59.43 ± 1.71	77.42

**Table 5:** Deployment-oriented complexity and inference-efficiency comparison of the evaluated backbone architectures.

Model	Params (M)	FLOPs (G)	FP32 Footprint (MB)	Latency (ms)	Peak Memory (GB)	Mean Macro-F1 (%)
ConvNeXt-T	27.82	4.47	106.1	5.3 ± 0.2	1.38	93.12
Swin-T	27.52	4.50	105.0	6.1 ± 0.3	1.42	90.21
DenseNet121	6.96	2.88	26.6	3.4 ± 0.1	0.82	86.07
MobileNetV3-Large	4.20	0.22	16.0	1.9 ± 0.1	0.48	77.42
ViT-B/16	85.80	17.58	327.3	15.2 ± 0.4	2.82	92.81
DeiT-B/16	85.80	17.58	327.3	14.8 ± 0.3	2.79	92.39

**Table 6:** Per-class recall (%) on Poly-Detail, aggregated across all five folds. The rightmost column reports the macro-average recall, reproduced from Table 4 for convenience.

Model	Intact	Crack	Broken	Surface-level	Macro
ConvNeXt-T	92.00	85.00	86.00	76.26	84.81
ViT-B/16	91.00	84.00	85.00	75.49	83.87
DeiT-B/16	90.00	83.00	84.00	73.29	82.57
Swin-T	88.00	78.00	80.00	66.80	78.20
DenseNet121	91.00	72.00	74.00	44.80	70.45
MobileNetV3	83.00	54.51	59.49	44.29	60.32

**Table 7:** Comparison with recent electroluminescence (EL)-based photovoltaic (PV) defect classification studies. CNN: convolutional neural network; CA: channel attention; GAN: generative adversarial network.

Study	Dataset	Classes	Best Backbone	Best Reported	Evaluation
Deitsch et al. [8]	ELPV (public)	4-grade defect	CNN (VGG-style)	88.42% accuracy	single split
Wang et al. [9]	ELPV + private	binary/multi	ResNet152–Xception + CA	96.17/92.13% accuracy.	single split
Akram et al. [18]	Private + ELPV	binary	Light CNN (custom)	93.02% accuracy.	single split
Tang1 et al. [9]	Private	multi-class	ResNet50 + GAN aug.	-	single split
Karakan [20]	Private mono + poly	3 (intact/ cracked/broken)	SqueezeNet	97.82/96.29% accuracy. (mono/poly)	single split, rot. aug.
Tella et al. [21]	ELPV	4/binary	ResNet18/voting ensemble	73.02/68.36% accuracy.	single split
Al-Otum [22]	ELPV (4/8-class)	4/8	LwNet (custom)	96.2% accuracy. (8-class)	single split
Ebied et al. [23]	Mono + poly	4 × 2	ResNet152 + GAN + dyn. thresh.	90.13% accuracy.	single split
Drir et al. [24]	Private (9 defects)	9	CNN-EML hybrid	—	single split
Our Study	Private mono + poly (20K cells)	5 tasks (2/2/3/2/4)	ConvNeXt-T	99.60/98.50/82.56/84.94% F1-macro	Stratified 5-fold CV

## Data Availability

The raw POLY electroluminescence images cannot be publicly redistributed because of institutional and industrial data-sharing restrictions. To support reproducibility within these constraints, the authors can provide the train/validation/test fold indices, group identifiers used for splitting, preprocessing and augmentation configuration, training hyperparameters, per-fold result files, and figure-generation scripts upon reasonable request, subject to institutional approval.

## References

[1] Nijsse F.J.M.M., Mercure J.-F., Ameli N., Larosa F., Kothari S., Rickman J., Vercoulen P., Pollitt H. (2023). The momentum of the solar energy transition. Nat. Commun..

[2] Yadav A.S., Agrawal V. (2018). Renewable Energy Sources: An Application Guide.

[3] Karaağaç M.O., Ergün A., Arslan O., Kayfeci M. (2023). Introduction to solar panels. Handbook of Thermal Management Systems.

[4] Maziuk M., Jasińska L., Domaradzki J., Chodasewicz P. (2023). Imaging methods of detecting defects in photovoltaic solar cells and modules: A survey. Metrol. Meas. Syst..

[5] Puranik V.E., Kumar R., Gupta R. (2023). Progress in module-level quantitative electroluminescence imaging of crystalline silicon PV module: A review. Sol. Energy.

[6] Jahn U., Herz M., Köntges M., Parlevliet D., Paggi M., Tsanakas I., Stein J.S., Berger K.A., Ranta S., French R.H. (2018). Review on Infrared and Electroluminescence Imaging for PV Field Applications.

[7] Potthoff T., Bothe K., Eitner U., Hinken D., Köntges M. (2010). Detection of the voltage distribution in photovoltaic modules by electroluminescence imaging. Prog. Photovolt. Res. Appl..

[8] Deitsch S., Christlein V., Berger S., Buerhop-Lutz C., Maier A., Gallwitz F., Riess C. (2019). Automatic classification of defective photovoltaic module cells in electroluminescence images. Sol. Energy.

[9] Wang J., Bi L., Sun P., Jiao X., Ma X., Lei X., Luo Y. (2022). Deep-learning-based automatic detection of photovoltaic cell defects in electroluminescence images. Sensors.

[10] Chen H., Pang Y., Hu Q., Liu K. (2020). Solar cell surface defect inspection based on multispectral convolutional neural network. J. Intell. Manuf..

[11] Han H., Gao C., Zhao Y., Liao S., Tang L., Li X. (2020). Polycrystalline silicon wafer defect segmentation based on deep convolutional neural networks. Pattern Recognit. Lett..

[12] Deitsch S., Buerhop-Lutz C., Sovetkin E., Steland A., Maier A., Gallwitz F., Riess C. (2018). Segmentation of photovoltaic module cells in electroluminescence images. arXiv.

[13] Dosovitskiy A., Beyer L., Kolesnikov A., Weissenborn D., Zhai X., Unterthiner T., Dehghani M., Minderer M., Heigold G., Gelly S. (2020). An image is worth 16×16 words: Transformers for image recognition at scale. arXiv.

[14] Carion N., Massa F., Synnaeve G., Usunier N., Kirillov A., Zagoruyko S., Vedaldi A., Bischof H., Brox T., Frahm J.M. (2020). End-to-End Object Detection with Transformers. Computer Vision—ECCV 2020.

[15] Liu Z., Lin Y., Cao Y., Hu H., Wei Y., Zhang Z., Lin S., Guo B. (2021). Swin Transformer: Hierarchical vision transformer using shifted windows. 2021 IEEE/CVF International Conference on Computer Vision (ICCV).

[16] Xie E., Wang W., Yu Z., Anandkumar A., Alvarez J.M., Luo P. (2021). SegFormer: Simple and efficient design for semantic segmentation with transformers. Adv. Neural Inf. Process. Syst..

[17] Mehta S., Rastegari M. (2021). MobileViT: Light-weight, general-purpose, and mobile-friendly vision transformer. arXiv.

[18] Akram M.W., Li G., Jin Y., Chen X., Zhu C., Zhao X., Khaliq A., Faheem M., Ahmad A. (2019). CNN based automatic detection of photovoltaic cell defects in electroluminescence images. Energy.

[19] Tang W., Yang Q., Xiong K., Yan W. (2020). Deep learning based automatic defect identification of photovoltaic module using electroluminescence images. Sol. Energy.

[20] Karakan A. (2025). Detection of defective solar panel cells in electroluminescence images with deep learning. Sustainability.

[21] Tella H., Hussein A., Rehman S., Liu B., Balghonaim A., Mohandes M. (2025). Solar photovoltaic panel cells defects classification using deep learning ensemble methods. Case Stud. Therm. Eng..

[22] Al-Otum H.M. (2024). Classification of anomalies in electroluminescence images of solar PV modules using CNN-based deep learning. Sol. Energy.

[23] Ebied M.A., Munshi A., Alhuzali S.A., El-sotouhy M.M., Shehta A.I., Elborlsy M.S. (2025). Advanced deep learning modeling to enhance detection of defective photovoltaic cells in electroluminescence images. Sci. Rep..

[24] Drir N., Chekired F., Mellit A., Blasuttigh N. (2025). Hybrid CNN-EML model for fault diagnosis in electroluminescence images of photovoltaic cells. Renew. Energy.

[25] Aktas M., Dogan F., Türkoglu I. (2026). A hybrid CNN–Transformer approach for photovoltaic cell defect classification using electroluminescence imaging. Sensors.

[26] Huang G., Liu Z., van der Maaten L., Weinberger K.Q. (2017). Densely connected convolutional networks. 2017 IEEE Conference on Computer Vision and Pattern Recognition (CVPR).

[27] Howard A., Sandler M., Chu G., Chen L.-C., Chen B., Tan M., Wang W., Zhu Y., Pang R., Vasudevan V. (2019). Searching for MobileNetV3. 2019 IEEE/CVF International Conference on Computer Vision (ICCV).

[28] Touvron H., Cord M., Douze M., Massa F., Sablayrolles A., Jégou H. (2021). Training data-efficient image transformers & distillation through attention. Proceedings of the 38th International Conference on Machine Learning (ICML 2021).

[29] Liu Z., Mao H., Wu C.-Y., Feichtenhofer C., Darrell T., Xie S. (2022). A ConvNet for the 2020s. 2022 IEEE/CVF Conference on Computer Vision and Pattern Recognition (CVPR).

[30] Russakovsky O., Deng J., Su H., Krause J., Satheesh S., Ma S., Huang Z., Karpathy A., Khosla A., Bernstein M. (2015). ImageNet Large Scale Visual Recognition Challenge. Int. J.-Comput. Vis..

[31] Zhang H., Cissé M., Dauphin Y.N., Lopez-Paz D. (2017). mixup: Beyond empirical risk minimization. arXiv.

[32] Szegedy C., Vanhoucke V., Ioffe S., Shlens J., Wojna Z. (2016). Rethinking the Inception architecture for computer vision. 2016 IEEE Conference on Computer Vision and Pattern Recognition (CVPR).

[33] Loshchilov I., Hutter F. (2017). Decoupled weight decay regularization. arXiv.

[34] Guo C., Pleiss G., Sun Y., Weinberger K.Q. (2017). On calibration of modern neural networks. Proceedings of the 34th International Conference on Machine Learning.

[35] Pedregosa F., Varoquaux G., Gramfort A., Michel V., Thirion B., Grisel O., Blondel M., Prettenhofer P., Weiss R., Dubourg V. (2011). Scikit-learn: Machine learning in Python. J. Mach. Learn. Res..

